# Safeguarding mothers and newborns: the urgent need to address hepatitis during pregnancy

**DOI:** 10.1186/s12916-023-03006-2

**Published:** 2023-08-02

**Authors:** BMC Medicine

**Affiliations:** The Campus, 4 Crinan Street, London, UK

“We are not waiting”—Under this central theme, we marked the annual Hepatitis Awareness Day on July 28th. It is crucial to emphasize this statement for the vulnerable populations affected: expectant mothers and newborn babies. They cannot afford to wait for hepatitis screening, treatment, and newborn vaccination. These individuals face unique risks of vertical (mother-to-child) transmission of the virus, which is often overlooked but is a critical issue concerning hepatitis infection during pregnancy.

This Editorial aims to shed light on managing viral hepatitis during pregnancy, stressing the significant importance of early testing, timely treatment, and vaccination in safeguarding the health of both mother and newborn.

According to the latest WHO global hepatitis report, hepatitis viruses (A, B, C, D, and E) cause 1.34 million deaths yearly due to liver complications. During pregnancy, viral hepatitis has significant consequences for mothers and infants. While hepatitis A, B, and C are the most common types (the ABCs of Viral Hepatitis), each virus presents unique challenges in pregnancy management.

Hepatitis viruses can cause acute infections during pregnancy, but HAV and HEV have the most severe complications and are common in low- and middle-income countries due to contaminated drinking water. HAV infection can lead to preterm labor, while HEV infection acquired in the second or third trimester carries substantial risks of morbidity and mortality.

While acute hepatitis is a short-term viral infection with sudden onset symptoms, typically resolving independently, chronic hepatitis is a long-term infection that persists for more than 6 months, potentially leading to liver damage and complications. For acute hepatitis, the main treatment approach involves supportive care, such as providing hydration and administering antiemetics. Liver transplantation may be necessary for severe cases, while vaccination (against HAV) and improved sanitation play vital roles in reducing maternal and fetal morbidity.

While acute viral hepatitis can occur during pregnancy, the primary focus lies on managing pregnant women with chronic viral hepatitis who were infected before entering pregnancy, which is a more prevalent concern. Worldwide, HBV, HCV, and HDV are associated with chronic infections affecting 325 million people, contributing to 96% of hepatitis-related mortality. However, there are notable regional variations in the burden of chronic hepatitis: based on a recent study, the combined pooled prevalence of HBV and HCV in pregnant women in low- and middle-income countries was up to 6.6% and 2.7%, respectively, in contrast to global prevalence rates of 4.8% and 1.0%, indicating that low- and middle-income countries are disproportionately affected.

Chronic viral infections are linked to pregnancy complications such as gestational diabetes for HBV infection and intrahepatic cholestasis of pregnancy for HCV infection and pose a significant risk of vertical transmission. Preventing transmission presents a substantial challenge. Globally, chronic HBV infection is of utmost concern due to the high number of HBsAg-positive women of childbearing age and the substantial risk of vertical transmission in the absence of prevention strategies. Therefore, early screening to identify infected mothers, antiviral therapy for those with a high viral load, and timely administration of HBV vaccine and hepatitis B immune globulin (HBIG) to infants is crucial for preventing HBV and HDV transmission. The Centers for Disease Control and Prevention recommends HBV screening for all pregnant people during each pregnancy, preferably in the first trimester, regardless of vaccination status or history of testing. In addition, healthcare providers should be vaccinated against HBV and tested for HCV after potential exposure.

Optimal and timely management of pregnant women and infants is vital to global efforts aimed at eliminating viral hepatitis. As no vaccine is available to protect against HCV, the focus is on identifying infected women, minimizing obstetrical contributions to perinatal transmission risks, and closely monitoring infants. Region-specific strategies, supported by national elimination plans and sufficient resources, are necessary to address the epidemiology and disease burden effectively. For instance, the TiP-HepC Registry project, led by the Coalition for Global Hepatitis Elimination and supported by the Centers for Disease Control and Prevention (CDC), aims to consolidate existing data and engage stakeholders to make informed decisions regarding the treatment of HCV during pregnancy. Another example is the WHO’s Triple Elimination Initiative, which urges countries to commit to eliminating the transmission of HIV, syphilis, and HBV from mother to child, promoting integrated service delivery.

Certainly, managing viral hepatitis during pregnancy requires special consideration. However, raising awareness among potentially infected individuals and sharing the ABCs of Viral Hepatitis is probably even more critical due to the risk of vertical transmission. Often, women are unaware of their infection and can pass it to their infants during childbirth, causing long-term liver damage, liver cancer, and cirrhosis: According to the latest WHO report, only 9% of those living with HBV infection knew their status. Therefore, the CDC recommends that all adults undergo testing for hepatitis B and C at least once in their lifetime, with pregnant women requiring testing during each pregnancy, as it is the only reliable method to determine hepatitis status.

Hepatitis in pregnancy revolves around the risk of vertical transmission, but interventions exist to prevent or reduce negative outcomes. The WHO’s 2016 strategy on viral hepatitis set an ambitious goal to eliminate it as a public health issue by 2030, targeting a 90% reduction in incidence and a 65% reduction in mortality compared to 2015. While England has already succeeded in meeting the new WHO targets for eliminating mother-to-child transmission of hepatitis B, achieving over 90% infant HBV vaccination coverage, hepatitis C among pregnant women has risen tenfold over the past 20 years in the USA, emphasizing the importance of universal screening for HCV infection in pregnancy. The urgency of addressing viral hepatitis during pregnancy cannot be underestimated: It cannot wait, and it is a responsibility we must all take seriously to ensure a healthier future for both mothers and their newborns.

This Editorial emphasizes the urgent need to address hepatitis during pregnancy on Hepatitis Awareness Day. BMC Medicine has an open Collection called “Maternal Factors during Pregnancy Influencing Maternal, Fetal, and Childhood Outcomes”, which welcomes exceptional contributions in these areas.

## Data Availability

Not applicable.

